# Heart failure among Indigenous Australians: a systematic review

**DOI:** 10.1186/1471-2261-12-99

**Published:** 2012-11-01

**Authors:** John A Woods, Judith M Katzenellenbogen, Patricia M Davidson, Sandra C Thompson

**Affiliations:** 1 Combined Universities Centre for Rural Health, PO Box 109, Geraldton, WA, 6531, Australia; 2 School of Population Health, University of Western Australia University of Western Australia, M431, 35 Stirling Highway, Crawley, WA, 6009, Australia; 3 Curtin Health Innovation Research Institute, Centre for International Health, Curtin University of Technology, GPO Box U1987, Perth, WA, 6845, Australia; 4 Faculty of Nursing, Midwifery and Health, University of Technology Sydney, PO Box 123, Broadway, NSW, 2007, Australia

**Keywords:** Heart failure, Australia, Indigenous, Aboriginal, Torres Strait Islander, Cardiac failure, Cardiovascular, Heart disease

## Abstract

**Background:**

Cardiovascular diseases contribute substantially to the poor health and reduced life expectancy of Indigenous Australians. Heart failure is a common, disabling, progressive and costly complication of these disorders. The epidemiology of heart failure and the adequacy of relevant health service provision in Indigenous Australians are not well delineated.

**Methods:**

A systematic search of the electronic databases PubMed, Embase, Web of Science, Cinahl Plus, Informit and Google Scholar was undertaken in April 2012 for peer-reviewed journal articles relevant to the topic of heart failure in Indigenous Australians. Additionally, a website search was done to identify other pertinent publications, particularly government reports.

**Results:**

There was a paucity of relevant peer-reviewed research, and government reports dominated the results. Ten journal articles, 1 published conference abstract and 10 reports were eligible for inclusion. Indigenous Australians reportedly have higher morbidity and mortality from heart failure than their non-Indigenous counterparts (age-standardised prevalence ratio 1.7; age-standardised hospital separation ratio ≥3; crude per capita hospital expenditure ratio 1.58; age-adjusted mortality ratio >2). Despite the evident disproportionate burden of heart failure in Indigenous Australians, the accuracy of estimation from administrative data is limited by poor indigenous identification, inadequate case ascertainment and exclusion of younger subjects from mortality statistics. A recent journal article specifically documented a high prevalence of heart failure in Central Australian Aboriginal adults (5.3%), noting frequent undiagnosed disease. One study examined barriers to health service provision for Indigenous Australians in the context of heart failure.

**Conclusions:**

Despite the shortcomings of available published data, it is clear that Indigenous Australians have an excess burden of heart failure. Emerging data suggest that undiagnosed cases may be common in this population. In order to optimise management and to inform policy, high quality research on heart failure in Indigenous Australians is required to delineate accurate epidemiological indicators and to appraise health service provision.

## Background

Indigenous Australians are known to suffer poorer health and lower life expectancy than their non-Indigenous counterparts. This life expectancy gap is attributable substantially to chronic conditions such as cardiovascular disease [1].

Heart failure (HF; synonyms: congestive heart failure, cardiac failure) is a ‘complex and lethal clinical syndrome’ [2] in which the heart is unable to provide blood flow adequate for the body’s metabolic needs [3]. Although HF is traditionally conceptualised as an impairment of the heart’s ability to pump sufficient blood into the circulation during systole, it is now recognised that left ventricular ejection fraction, a measure of systolic function, is preserved in many cases [4], and that this pathophysiological heterogeneity of HF may be influenced by the spectrum of underlying causes [5].

The antecedents of HF, especially hypertension [6,7] and coronary heart disease including myocardial infarction (MI) [8,9], are disproportionately common among Indigenous Australians. Importantly, these conditions tend to become manifest at a younger age than among non-Indigenous persons, resulting in a much greater burden of disease, and contributing to greater levels of disability in Indigenous populations [1]. Renal disease, which clusters with features of the metabolic syndrome as a predictor of cardiac illnesses, is prevalent at extremely high levels in some Indigenous populations [10,11]. Diabetes, frequently associated with this cluster of cardiovascular conditions, also occurs at a much higher rate among indigenous Australians [12], and may independently predispose to HF [13]. Moreover, Indigenous Australians are known to suffer an exceptionally high incidence of rheumatic fever, with the potential sequelae of chronic rheumatic heart disease and HF [14].

Regardless of the underlying cause, HF is an important cardiovascular problem in its own right, being associated with substantial disability, impaired quality of life and diminished survival [15]. Further, timely identification of HF with institution of evidence-based interventions diminishes symptoms and potentially prolongs life [16,17]. However, the epidemiological indicators of HF among Indigenous Australians are poorly delineated. Although administrative data on HF in the Australian population are published regularly, particularly by the Australian Institute of Health and Welfare (AIHW), the accuracy of such administrative data, and the validity of epidemiological comparisons between Indigenous and non-Indigenous populations derived from these data are fraught, doubly in this instance given that there are caveats on both ascertainment of HF [18,19] and identification of Indigenous status [20]. Further, administrative data are generally not person-based, for example, re-admissions of the same patient cannot be distinguished [21]. Consequently, quality research specifically addressing HF in the Indigenous population is needed to verify indicator measures derived from administrative data.

Personal and psychosocial perspectives, based on qualitative research, are also important for understanding the effects of HF in the Indigenous population. Such information is a valuable adjunct to quantitative data in estimating the impact of the disease and to inform planning.

This review specifically explores publicly available information on HF in the Australian Indigenous population through: (1) a systematic search of the peer-reviewed literature and (2) a review of reports published on this topic based on analyses of administrative data by Australian federal and state/territory governments.

## Methods

### Search of peer-reviewed journal databases

A systematic search of the electronic databases PubMed, Embase, Scopus, Web of Science, Cinahl Plus, Informit and Google Scholar was undertaken, using search terms that comprised: (i) subject headings, specified by the respective databases related to heart failure or to Indigenous status (for example Medical Subject Heading [MeSH] in PubMed), and (ii) other synonyms for HF and Indigenous status, either listed by the electronic databases or generated by the authors. The search retrieved articles containing one or more terms relevant to both heart failure and Australian Indigenous (Aboriginal or Torres Strait Islander) status. A full list of search terms used is provided below (Additional file 1: Table S1). The search was restricted to articles published from 1990 onwards and to English language publications. Duplicates were identified and removed by automated search and manual scrutiny of the remaining list of citations.

The search strategy was modified for Google Scholar. Given the limited Boolean syntactical capacity of the Google Scholar search engine [22], a search with fewer, more general search terms was done. This retrieved a large number of citations (about 3000) including articles not elsewhere identified that contained ‘heart failure’ as a phrase in the full text, even if this was not present in the title, abstracts or keywords. The first 50 citations were screened manually for relevance and to ensure non-duplication of citations from other databases.

### Search of websites for relevant reports

The Australian Government and state/territory Department of Health websites were searched from within the Google interface or using departmental website internal search engines for documents with the terms ‘Aboriginal or Indigenous or heart failure or cardiac failure’. The Australian Institute of Health and Welfare (AIHW) website was independently searched for relevant articles as was the Australian Indigenous Health *Info*Net website, a clearinghouse specifically devoted to reports and information pertaining to health of Indigenous Australians. References lists from articles and reports included in the search were also checked for relevant publications.

### Inclusion criteria

Studies were considered for inclusion if they dealt with the Australian Indigenous population and included post-1990 data or qualitative evidence on one or more pre-defined heart failure-related issues related to (1) prevalence or incidence, either population-based or within clinical groups or clinical service settings (such as acute coronary syndrome cohorts); (2) aetiology, risk factors or clinical presentation and pathophysiology; (3) co-morbidities; (4) mortality & survival; (5) quality of life; (6) therapeutic interventions; (7) health service utilisation (including medication adherence, primary care attendances, hospitalisations, cardiac rehabilitation); (8) health service delivery issues (including needs, access and barriers); and (9) costs related to HF diagnosis and care.

One author (JW) screened all of the references (n=127) by title and abstract. A large number of references were deemed not relevant on the basis of this initial screening. The criteria for exclusion on this preliminary screening (Table 1) were checked and accepted by other authors (JK and ST). The remaining references (n = 51) were reviewed jointly by two authors (JW and JK).

**Table 1:** Criteria for Exclusion based on Title/Abstract Alone

	**N**
Indigenous other than Australian Aborigines or Torres Strait Islander peoples:	
1. Non-Australian Indigenous human subjects	**59**
2. ‘Indigenous’ botanicals	**11**
3. Other non-anthropological meanings of ‘indigenous’ (e.g., as synonym for ‘intrinsic’)	**10**
Review articles – no original data	**6**
Duplicate data	**2**
Case report data only	**6**
**TOTAL**	**94**

Although at the outset it was not proposed to include conference abstracts, in view of the scarcity of published information, one abstract was ultimately included to reflect work/research as yet unpublished.

## Results

**Figure 1:**
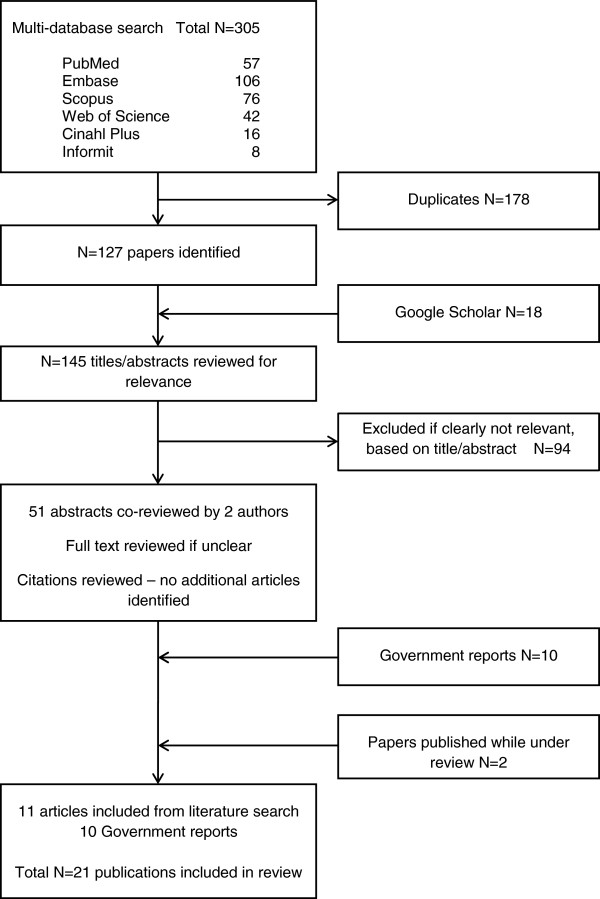
Flowchart.

Of 145 journal articles or conferences abstracts identified (Figure 1), 94 were excluded on the basis of preliminary screening (Table 1). Of the 51 remaining papers reviewed by two authors, 9 were eligible for inclusion with a further two eligible articles published while the manuscript was under review (Table 2). Ten reports were also included (Table 3).

**Table 2:** Peer-reviewed journals

**Author(s)/year Publication Type**	**Study Population and Time Period**	**Methods**	**Key finding(s) pertinent to heart failure**	**Validity and generalisability issues (including Indigenous identification)**
**1. Prevalence or incidence, either population-based or within clinical groups or clinical service settings**				
Coory et al. (2005) [23] Original article	Queensland patients hospitalised with MI in public sector N=14683, Indigenous=558	Design: Retrospective cohort study (I year follow-up)	HF more commonly a concurrent co-morbidity among Indigenous than non-Indigenous identified patients (age-adjusted RR 1.64; CI 1.35-2.00)	• HF one of several co-morbidities assessed; not an endpoint of study.
		Data source: linked hospital data		
				• No adjustment for administrative under-identification of Indigenous status
	Period: Admitted for MI 1998-2002	Outcome: Revascularisation		
Katzenellenbogen et al. (2012) [24] Original article	All WA patients hospitalised with non-fatal first-ever MI N=7480, Indigenous=532	Design: retrospective cohort study (2 year follow-up) Data source: Linked administrative data	HF more commonly a concurrent or past co-morbidity among Indigenous than non-Indigenous identified patients (males: 17% vs. 13%; p=0.018; females: 31% vs. 22%; p=0.003)	• HF one of several co-morbidities assessed; not an endpoint of study.
				• Indigenous status based on ever-identification in hospital or death records
	Period: Admitted Jan 2000-Dec 2004	Outcome: recurrent MI/CVD death		
				• Crude HF prevalence, no age adjustment thus underestimate of disparity
McGrady et al. (2012) [25] Original Article	Consenting Aboriginal adults (>18 years) residing in one of six Central Australian communities, (Alice Springs, Town Camp or remote) N=436 (mean age 44 years)	Design: cross-sectional clinical survey	HF detected in 5.3% (CI 3.2-7.5%); 65% of these no pre-existing HF diagnosis. ALVD present in a further 13% (CI 9.4-15.7%)	• Population-based study designed specifically to assess epidemiology of HF and risk factors among Central Australian Aboriginal adults.
		Data source: Clinical history & examination, anthropometry, echocardiogram, biochemistry		
				• Volunteer participants – representativeness uncertain
	Period: Assessment May 2008 - Nov 2009	Outcome: presence of HF or ALVD		
				• No non-Indigenous comparison group
**2. Aetiology, risk factors, clinical presentation and pathophysiology**				
Einsiedel et al. (2012) [26] Original article	Indigenous adults (n=89) admitted to a single general hospital with bronchiectasis and known HTLV-1 serological status	Design: Retrospective cohort study Data source: Hospital records	HF (35% versus 11%; p=0.013) more common in human HTLV-1 seropositive than HTLV-1 seronegative subjects.	• HF one of several complications assessed
				• Indigenous status identified from medical records
				• Comparison not age-adjusted,
				• however mean age essentially identical in both groups.
				• Population restricted
	Period: Jan 2000-Dec 2006	Outcome: bronchiectasis outcomes		• to Central Australia, majority ‘remote’ residence (61%)
Greaney (2010) [27] Original article	Indigenous patients with symptomatic HF referred to a heart rehabilitation program in Far North Queensland (n=101)	Design: Descriptive clinical survey	57% had normal systolic function	• Indigenous status identification not explicit
		Data source: Hospital and echocardiograph records		• No non-Indigenous comparison group
	Period: April 2005-Jan 2008	Outcome: Proportion with normal systolic function		
McGrady et al. (2012) [25] Original article	Aboriginal adult volunteers (>18 years) residing in six Central Australian communities, N=436 (mean age 44 years)	Design: cross-sectional clinical survey	Age & sex-adjusted odds ratio for HF: CAD (9.6, p<0.001)	• Population-based study designed specifically to assess epidemiology of HF and risk factors among Central Australian Aboriginal adults.
		Data source: Clinical history & examination, anthropometry, echocardiogram, biochemistry		
			DM (5.4, p=0.002)	
			HT (4.8, p=0.006)	
	Period: Assessment May 2008 - Nov 2009	Outcome: presence of HF or ALVD	Obesity (2.9, p=0.022)	
				• Volunteer participants – representativeness uncertain
			RHD history (5.6, p=0.001) 39% of HF cases had preserved ejection fraction	• No non-Indigenous comparison group
**3. Co-morbidities**				
McGrady et al. (2012) [25] Original Article	Aboriginal adult volunteers (>18 years) from six Central Australian communities, N=436 (mean age 44 years)	Design: cross-sectional clinical survey	Crude prevalence in HF cases: Diabetes 78% Hypertension 78% CAD 39% ARF/RHD 26%	• Population-based study designed specifically to assess epidemiology of HF and risk factors among Central Australian Aboriginal adults.
		Data source: Clinical history & examination, anthropometry, echocardiogram, biochemistry		
	Period: Assessment May 2008 - Nov 2009	Outcome: presence of HF or ALVD		• Volunteer participants – representativeness uncertain
				• No non-Indigenous comparison group
**4. Mortality & survival**				
Brown (2010) [28] Original article	Patients admitted to two NT hospitals with ACS (n=214 Indigenous, 278 non-Indigenous)	Design: Retrospective audit	Frequency of death attributed to HF was similar in both Indigenous (approx. 2.2%) and non-Indigenous (approx. 2.0%).	• Indigenous identification is relatively good in NT administrative records; no additional effort to improve identification
				• Sample size relatively small; Only 2 hospitals in sample
		Data source: Hospital records		
	Period: Admissions Jan 2001-Dec 2002; 2 year follow-up	Outcome: All-cause and CVD deaths		
Carapetis et al. (1999) [29] Original article	80 consecutive patients (70 Indigenous) with surgical valve replacement for RHD	Design: Cohort study	29 late deaths, 27 attributed to RHD, 12 of these were HF deaths (plus 1 due to ‘HF and pneumonia’)	• No comparison group.
		Data source: Hospital records		• Long calendar period of case acquisition (1964–1996) limits contemporary interpretability of prognosis.
	Period: Surgery 1964–1996; all patients followed up until Mar 1997	Outcome: Deaths		
Katzenellenbogen et al. (2011) [24] Original article	See Part 1. above	See Part 1. above	HF as a co-morbidity independently associated with about double the risk of composite outcome (recurrent AMI or death) in both Indigenous and non-Indigenous subjects	• HF was one of a number of demographic and co-morbidity variables in the model
**5. Quality of life**				
Nil				
**6. Therapeutic interventions**				
Nil				
**7. Health service utilisation (including medication adherence, outpatient attendances, hospitalisations, cardiac rehabilitation)**				
Bolton et al. (2011) [30] Conference abstract	Patients referred to inner-suburban AMS-controlled cardiology clinic	Design: Cross sectional survey	2 of 68 patients (3%) referred to an inner-suburban AMS-controlled cardiology clinic had HF.	• Encounter proportions without comparison group difficult to interpret
		Data source: Clinic records		
	Period: July 2009-2011	Outcome: Attendance, encounter proportions		
				• Uncertain generalisability to Australian Indigenous population.
Thomas et al. (1998) [31] Original article	Primary care (AHW and/or doctor) encounters in AMS clinic in Darwin, NT.	Design: Cross sectional survey	Proportion of encounters involving HF: 3.4% (95% CI 1.9-4.9) compared with 1.6% in national comparison data (AMTS)	• Comparison of encounter proportions difficult to interpret
		Data source: Clinic records		
				• Uncertain generalisability to Australian Indigenous population.
	Period: 2 separate study weeks 6 months apart: in Darwin’s wet season (Feb 1994) & in dry season (Aug 1994)	Outcome: Encounter proportions		
**8. Health service delivery issues (including needs, access and barriers)**				
Clark et al. (2007) [32] Original article	(sample n/a) Period: Jan 2004-Dec 2005	Design: Cross sectional geomapping survey	Highest prevalence of HF in areas with people aged >65 years and higher proportions of Indigenous people.	• No direct measure of HF prevalence (based on international prevalence data).
		Data source: Census data, international prevalence estimates		
				• Indigenous population distribution derived from Census data.
		AIHW-derived Indigenous HF prevalence estimates.		
				• Indigenous:non-Indigenous HF prevalence ratio estimated from AIHW HF mortality data.
		Outcome: Indirect measure of access to CR services		
			Geographical inequity in provision of HF specialist management programs, with limited access in rural areas.	
Aspin et al. (2012) [32] Original article	19 Indigenous subjects (age range 34–70) from Western Sydney or Aust Capital Territory: 11 had HF, with/without co-existing diabetes and/or COPD.	Design: Interviews with Aboriginal people with chronic disease, recruited via Aboriginal health services	Negative influences were poor access to culturally appropriate health services, dislocation from cultural support systems, racism, poor communication with health professionals and economic hardship Positive influences were strength drawn from being part of the Aboriginal community, regular ongoing access to primary care and a supportive family network	• Findings not specific to HF but reflect issues related to chronic disease care of HF prevalence (based on international prevalence data).
		Data source: n.a.		• Indigenous population distribution derived from Census data.
	Period: Jan 2004-Dec 2005	Outcome: Description of barriers and facilitators of access to care and support		
				• Indigenous: non-Indigenous HF prevalence ratio estimated from AIHW HF mortality data.
**9. Costs related to HF diagnosis and care**				
Nil				

*ACS:* acute coronary syndrome.

*AHW:* Aboriginal Health Worker.

*AIHW:* Australian Institute of Health and Welfare.

*ALVD:* Asymptomatic left ventricular dysfunction.

*AMS:* Aboriginal Medical Service.

*AMTS:* Australian Morbidity and Treatment Survey.

*CAD:* coronary artery disease.

*CI:* 95% confidence interval.

*COPD:* Chronic obstructive pulmonary disease.

*CR:* cardiac rehabilitation.

*CVD:* cardiovascular disease.

*DM:* diabetes mellitus.

*HF:* heart failure.

*HT:* hypertension.

*HTLV-1:* Human T-lymphotropic virus type 1.

*MI:* myocardial infarction.

*OR:* odds ratio.

*RHD:* rheumatic heart disease.

*RR:* risk ratio.

**Table 3:** Reports

**Author(s)/year**	**Population and Calendar period**	**Methods**	**Key findings pertinent to heart failure**	**Validity and generalizability issues (including Indigenous identification)**
**1. Prevalence or incidence, either population-based or within clinical groups or clinical service settings**				
NATSIHS Survey reported in Penm (2008) [33]	Whole of Australia Indigenous population (Residents in Very Remote areas not included in non-Indigenous NHS comparator group)	Design:	Standardised prevalence ratio of HF among Indigenous Australians 1.7 (males 1.9; females 1.6)	• Ascertainment of HF based on self-report; conflated with self-report of oedema.
		Cross-sectional survey		
		Data source: questionnaire of persons usually resident in private dwellings		
				• Comparator non-Indigenous data excluded subjects in Very Remote areas.
	Period: August 2004 to July 2005	Outcome: Self-reported health problems		• Low precision of SPR estimate, especially for males
				• Indigenous status according to self-identification in Census
**2. Aetiology, risk factors, clinical presentation and pathophysiology**				
Nil				
**3. Co-morbidities**				
Nil				
**4. Mortality & survival**				
Field (2003) [34]	SA, Qld, WA, NT population	Design: Descriptive study	Indigenous HF mortality rates almost threefold higher than non-Indigenous. Disproportionately high HF mortality among Indigenous males aged 55–64 years.	• Rates calculated for population aged ≥45 years only
		Data source: Administrative data (NMD)		
				• Inter-jurisdictional variation in Indigenous identification data quality
	Period: 1995–96 to 1997–98 and 1998–99 to 2000-01	Outcome: Deaths		
				• Inherent shortcomings of HF identification on death certificates
Penm (2008) [33]	SA, Qld, WA, NT population	Design: Descriptive study	Age-adjusted Indigenous HF mortality rates more than double non-Indigenous rates.	• Inter-jurisdictional variation in Indigenous identification data quality
	Period: 2002-05	Data source: Administrative data (NMD)	In 45–64 year age-group, mortality rate ratio 6.4.	
				• Inherent shortcomings of HF identification on death certificates
		Outcome: Deaths		
**5. Quality of life**				
Nil				
**6. Therapeutic interventions**				
Nil				
**7. Health service utilisation (including medication adherence, outpatient attendances, hospitalisations, cardiac rehabilitation)**				
**(a) Primary care attendances**				
BEACH Survey reported in AIHW (2008) [35]	GP practices Australia-wide	Design: Cross-sectional survey	Crude proportion of HF encounters lower among Indigenous (1.0/100, CI 0.6-1.3) than non-Indigenous patients (0.7, CI 0.7-0.8)	• Data difficult to interpret: not person-based (cannot identify recurrent attendances for the same person), estimates conflate differences in underlying morbidity with differences in service access and utilisation
	Period: 2002–03 to 2006-07			
		Data source: Written questionnaires (100 consecutive encounters from ~1000 participating GPs nationwide)		
			Age-standardised proportion of HF encounters higher for Indigenous patients (ratio 2.6)	
		Outcome: Indications for GP encounters		
				• No formal basis for Indigenous identification; patients not providing Indigenous status conflated with ‘non-Indigenous’
				• Imprecise estimates for Indigenous attendances
Beach Survey AIHW (2011) [36]	GPs Australia-wide	Design: Cross-sectional survey	Crude proportion of HF encounters lower among Indigenous (0.9/100, CI 0.6-1.2) than non-Indigenous patients (0.7, CI 0.7-0.7)	• Data difficult to interpret: not person-based (cannot identify recurrent attendances for the same person), estimates conflate differences in underlying morbidity with differences in service access and utilisation
	Period: April 2004-March 2005 to April 2008-March 2009			
		Data source: Written questionnaires (100 consecutive encounters from ~1000 participating GPs nationwide)		
			Age-standardised proportion of HF encounters higher for Indigenous patients (ratio 2.6)	
		Outcome: Indications for GP encounters		• No formal basis for Indigenous identification; patients not providing Indigenous status conflated with ‘non-Indigenous
				Imprecise estimates for Indigenous attendances
**(b) Hospitalisations**				
Nichol (1999) [37]	Patients admitted to Australian public and private hospitals	Design: Descriptive study	970 separations with principal diagnosis HF among indigenous; 39,305 Non-Indigenous	• Separation rate-ratio not provided
		Data source: Administrative data (NHMD)	Crude average length of hospital stay for ‘congestive heart failure) shorter for Indigenous than non-Indigenous patients (6.5 vs 9.4 days)	• Data not person-based: cannot identify recurrent separations for the same person
	Period:July 1995-June 1996	Outcome: Principal diagnosis reported for hospital separations		• Indigenous identification varies between jurisdictions, Indigenous identity likely under-identified at a single separation
				• Caveats of HF-related code as principal diagnosis
Field (2003) [34]	Patients admitted to SA and NT hospitals only	Design: Descriptive study	July 1998-June 2001 triennium: age-standardised separation rates (HF or hypertensive heart disease) higher among Indigenous than non-Indigenous patients (males: 1555/10^5^ vs 743/10^5^; females: 1579/10^5^ vs 541/10^5^)	• Rates calculated for population aged ≥45 years only
		Data source: Administrative data (NHMD)		
				• Data not person-based so cannot distinguish repeat recurrent separations for the same person.
	Period: 1995–96 to 1997–98 and 1998–99 to 2000-01	Outcome: Principal diagnosis reported for hospital separations		
			HF hospitalisation rates fell among both sexes, in both Indigenous and non-Indigenous populations, between 1995–98 and 1998–2001 triennia.	
				• Not nationwide data: SA/NT only.
				• Indigenous identity likely under-identified at a single separation.
AIHW (2008) [35]	Patients admitted to private (excluding NT) and public hospitals in NSW, Vic, Qld, WA, SA and NT.	Design: Descriptive study	Age-standardised hospital separation ratio (Indigenous:non-Indigenous) for HF 3.4.	• Data not person-based: cannot identify recurrent separations for the same person
		Data source: Administrative data (NHMD)	Average bed days for congestive heart failure 5.7 (Indigenous patients); 7.7 (non-Indigenous)	
				• Report restricted to jurisdictions with better Indigenous identification, however this varies between included jurisdictions, Indigenous identity likely under-identified at a single separation
	Period: July 2004 to June 2006	Outcome: Diagnoses reported for hospital separations		
AIHW (2011) [36]	Patients admitted to private (excluding NT) and public hospitals in NSW, Vic, Qld, WA, SA and NT.	Design: Descriptive study	Age-standardised hospital separation ratio (Indigenous:non-Indigenous) for HF 3.0.	• Data not person-based: cannot identify recurrent separations for the same person
		Data source: Administrative data (NHMD)		
	Period: July 2006 to June 2008	Outcome: Diagnoses reported for hospital separations	Average bed days for congestive heart failure 5.4 (Indigenous patients); 7.5 (non-Indigenous)	• Report restricted to jurisdictions with better Indigenous identification, however this varies between included jurisdictions, Indigenous identity likely under-identified at a single separation
Steering Committee (2011) [38]	Patients admitted to private (excluding NT) and public hospitals in NSW, Vic, Qld, WA, SA and NT.	Design: Descriptive study	Age-standardised hospital separation rates for congestive heart failure 6.1 (Indigenous) vs 2.0 (non-Indigenous)	• Data not person-based: cannot identify recurrent separations for the same person
		Data source: Administrative data (NHMD)		
	Period: 2008-09	Outcome: Diagnoses reported for hospital separations		• Indigenous identification varies between jurisdictions, Indigenous identity likely under-identified at a single separation
AIHW (2011) [39]	Patients admitted to public and private hospitals in all states and territories.	Design: Descriptive study	Crude hospital separation rates for congestive heart failure:	• Data from all states and territories.
		Data source: Administrative data (NHMD)	Indigenous: 2.8/1000 Non-Indigenous: 2.1/1000	• Data not person-based: cannot identify recurrent separations for the same person
	Period: 2008-2009	Outcome: Principal diagnosis reported for hospital separations	(Rate ratio: 1.33)	
				• Rates adjusted for Indigenous under-identification.
				• Crude rates only.
Bureau of Health Information (NSW) (2011) [40]	Patients >45 years admitted to public and private hospitals in NSW.	Design: Descriptive study	2% of ‘potentially avoidable’ HF admissions of patients occurred among patients identified as Aboriginal, with ‘2% of the NSW population’ considered to be Aboriginal.	• Data not person-based: cannot identify recurrent separations for the same person
		Data source: Administrative data (APDC)		
	Period: July 2009-June 2010	Outcome: ‘potentially avoidable’ admissions for specified conditions (including HF)		• Crude proportion only
				No adjustment for Indigenous under-identification in hospitalisation data
Bureau of Health Information (NSW) (2012) [41]	Patients >45 years with pre-existing record of HF hospitalisation admitted to public and private hospitals in NSW.	Design: Cohort study	Patients with pre-identified HF admitted on >1 occasion with HF during year of study were more likely to be Aboriginal (3%) than those with 0–1 HF admissions (2%)	• Person-based data
		Data source: Linked administrative data (APDC and mortality)		• Proportion of cohort identified as Aboriginal not stated
				No adjustment for Indigenous under-identification
	Period: July 2009-June 2010	Outcome: admissions and re-admissions		
**8. Health service delivery issues (including needs, access and barriers)**				
Nil				
**9. Costs related to HF diagnosis and care**				
AIHW (2011) [39]	Patients admitted to public and private hospitals in all states and territories.	Design: Descriptive study	For congestive heart failure, patients identified as Indigenous accounted for 3.9% of total expenditure for this condition. Expenditure on CHF hospitalisation per person:	• Data from all states and territories.
		Data source: Administrative data (NHMD)		• Indigenous identification varies between jurisdictions, Indigenous identity likely under-identified at a single separation
	Period: 2008-2009	Outcome: Expenditure on potentially preventable hospital separations	Indigenous $26.70	
			Non-Indigenous $16.90	
			(Indigenous:non-Indigenous expenditure ratio 1.58)	

*AIHW:* Australian Institute of Health and Welfare.

*APDC:* Admitted Patient Data Collection (New South Wales).

*BEACH:* Bettering the Evaluation and Care of Health.

*HF:* heart failure.

*NATSIHS:* National Aboriginal and Torres Strait Islander Health Survey.

*NHMD:* National Hospital Morbidity Database.

*NHS:* National Health Survey.

*NMD:* National Mortality Database.

### Prevalence or incidence, either population-based or within clinical groups or clinical service settings (such as acute coronary syndrome cohorts)

#### Peer-reviewed studies

There was only one peer-reviewed, population-based study of HF prevalence in the Australian Indigenous population, and none of incidence rates. McGrady and colleagues reported on the “Heart of the Heart” study, in which volunteering adult Aboriginal participants from 6 Central Australian communities were enrolled in 2008–9 for a comprehensive cardiac assessment which included a researcher-administered structured questionnaire (medical history, drugs, smoking, alcohol intake and HF symptoms), medical record review, anthropomorphic measures (waist circumference, height and weight), blood pressure, cardiac and lung auscultation and fluid status assessment by a study cardiologist, biochemistry (for assessment of renal function, random non-fasting blood glucose level, glycosylated haemoglobin (Hb_A1c_) and B-type natriuretic peptide (BNP) [25]. The prevalence of HF was 5.3% (95%CI 3.2-7.5%), with asymptomatic left ventricular dysfunction present in a further 13%. In 65% of those with HF detected, the disorder had not been previously diagnosed. The interpretability of the reported prevalence estimate in this study is limited by the absence of a non-Indigenous comparison group and uncertain representativeness of the participants in relation to the Central Australian Aboriginal community, although the age profile and prevalence of comorbidities are similar to findings of other whole-of-community medical record reviews.

Two retrospective cohort studies, both based on administrative linked data, reported the frequency of several co-morbidities, including HF, among Indigenous subjects with coronary heart disease. In a study of patients admitted to Queensland public hospitals with acute myocardial infarction MI [23], Indigenous subjects had a higher prevalence of HF as a concurrent comorbidity (age-adjusted risk-ratio 1.67; 95% CI 1.35-2.00). In another study of 28-day survivors of first-ever MI in Western Australia, current or 5-year history of HF was more common among Indigenous subjects than their non-Indigenous counterparts (males: 17% versus 13%; p=0.018; females: 31% versus 22%; p=0.003), even though the median age of Indigenous subjects was 13–14 years lower [24].

#### Government reports

The periodic National Aboriginal and Torres Strait Islander Health Survey (NATSIHS) provides data on self-reported health problems. In the 2004–05 NATSIHS, information was collected from a sample of 10,400 persons, constituting about 1 in 45 of the total Indigenous population [33]. Comparator non-Indigenous data were obtained from the 2004–05 National Health Survey (n=approximately 25,900), intended to be representative of Australian households (but excluding subjects from Very Remote locations, unlike the NATSIHS). Survey samples included only the usual residents of private dwellings. The presence of ‘oedema’ was considered synonymous with HF in the self-reported data. In this survey, the overall prevalence of self-reported HF or oedema among Indigenous Australians was 1.0%, with age-standardised prevalence estimates of 17.4/1000 (95% CI 6.3-28.5) in Indigenous males and 28.8/1000 (95% CI 17.0-40.7) in Indigenous females. The precision of these estimates is likely to be poor, with a relative standard error of 25-50% reported for the male estimate. The Indigenous to non-Indigenous age-standardised prevalence ratio was 1.7 overall (males 1.9; females 1.6, not statistically significant for either sex, but significant for both sexes combined) [33].

### Aetiology, risk factors, clinical presentation and pathophysiology

In the recent report by McGrady and colleagues, the key risk factors associated with HF in a Central Australian Aboriginal population were coronary heart disease, hypertension, diabetes mellitus, obesity, and a history of acute rheumatic fever or rheumatic heart disease (Table 2). Diabetes, hypertension and obesity (individually or in combination) were contributing factors in 48% of HF cases and 79% of those with asymptomatic left ventricular dysfunction, while 9% and 13% of these cases respectively had no clearly identified contributory risk factor [25]. Although the likely antecedents of HF in the Indigenous population (particularly coronary and rheumatic heart disease) are well discussed in the literature, HF was otherwise only mentioned incidentally in several studies of Indigenous Australians as a fatal complication (see below) of coronary [28] or rheumatic heart disease [29].

No studies quantified the contribution of pulmonary disease to HF in Indigenous Australians, or linked chronic obstructive pulmonary disease with HF in this population. However, co-morbid HF was reported in one retrospective cohort study of the role of human T-lymphotropic virus-1 (HTLV-1) infection in determining outcomes among Central Australian Indigenous adult subjects who had been hospitalised with bronchiectasis (n=89) [26]. HF was present in 22 patients and cor pulmonale was diagnosed in 11. HTLV-1 seropositivity was associated with worse outcomes. In addition to an increased likelihood of adverse radiographic indicators of pulmonary disease and increased bronchiectasis-specific mortality in HTLV-1 seropositive patients, HF (35% versus 11%; p=0.013) and cor pulmonale specifically (19% versus 3%; p=0.023) were both more common in HTLV-1 seropositive than HTLV-1 seronegative subjects.

The frequency of HF with preserved ejection fraction was only addressed in two published studies. In a study of HF patients identifying as Indigenous referred for echocardiography in north Queensland [27], the reported proportion with preserved ejection fraction (57%) was considered to be ‘at the upper end of that expected’ from studies of other populations. However, the study did not provide non-Indigenous comparison data. In the Central Australian study, 61% of those with HF had impaired left ventricular systolic function (ejection fraction <50%) and 39% had HF with preserved ejection fraction [25].

### Co-morbidities

Despite the known endemicity of chronic diseases such as diabetes and renal dysfunction among Indigenous Australians, the only publication which dealt specifically with co-morbidities for HF per se in an Indigenous population was the work of McGrady and colleagues. They reported the following comorbidities being significantly more prevalent in HF cases than non-cases in the Central Australian Aboriginal adult population: CAD (39%), Diabetes (78%), hypertension (78%) and ARF/RHD (26%) [25].

### Mortality & survival

#### Peer-reviewed studies

The peer-reviewed literature provided no population-based data on HF mortality among Indigenous Australians. However, deaths attributed to HF have been examined in the context of coronary heart disease and rheumatic heart disease (RHD). The Central Australian Secondary Prevention of Acute Coronary Syndromes (CASPA) Study was a retrospective audit of patients hospitalised with ACS in the Northern Territory [28]. After 2-year follow-up, the crude frequency of deaths attributed to HF was similar in Indigenous and non-Indigenous groups. However, Indigenous subjects had a substantially lower mean age on admission (50.1 vs. 59.3 years; p<0.001), despite which they were significantly more likely than non-Indigenous subjects to have died from any cause (30.0% vs. 17.8%; p=0.002). In the previously cited WA cohort of first-ever MI survivors [24], HF as a co-morbidity was independently associated with about double the risk of either recurrent AMI admission or cardiovascular death in Indigenous and non-Indigenous subjects.

Carapetis et al. [29] examined the outcome of cardiac valve replacement in subjects with RHD in northern Australia. In this retrospective chart review with some prospective follow-up of 80 consecutive patients (70 of whom were Indigenous), there were 29 late deaths, including 27 considered consequent to the RHD, 12 of which were attributed to HF and 1 to ‘pneumonia with HF’. The ages of the patients at the time of death were not reported.

#### Government reports

In a report specifically addressing HF, *Heart failure…what of the future?*[34], the AIHW provided National Mortality Database (NMD) data from South Australia, Western Australia, Queensland and the Northern Territory only, due to poor Indigenous identification in administrative data in other jurisdictions. Death rates attributed to HF were reported to have remained ‘fairly static’ between 1995-96/1997-98 and 1998-99/2000-01 among male and female Indigenous and non-Indigenous populations. However, age-standardised death rates (45 years and over) in the period 1998–99 to 2000–01 where HF or hypertensive heart disease was certified as the underlying cause were nearly threefold higher in the Indigenous population (173 per 100,000 males and 160 per 100,000 females) than in the non-Indigenous population (60 per 100,000 males and 66 per 100,000 females). The study found that there was disproportionately high mortality among middle-aged Indigenous males. Indeed, the death rate among 55–64 year old males (47 per 100,000) was higher than among 65–74 year olds (34 per 100,000), although the rate rose markedly in the Indigenous male population aged 75 years and over.

In a more recently published AIHW report, again based on NMD data from the same four jurisdictions for the period 2002–04 [33], age-adjusted mortality attributed to HF was estimated to be over twice as high in Indigenous as non-Indigenous Australians overall, and 6.4 times as high in the 45–64 year age group. The overall standardised HF mortality ratio was more accentuated for the female (2.4) than the male Indigenous population (2.0).

### Quality of life

There were no studies or reports dealing specifically with quality of life in HF *per se* among Indigenous Australians.

Therapeutic interventions

There were no studies or reports dealing specifically with therapeutic interventions for HF tailored and targeted for Indigenous Australians.

### Health service utilisation (including medication adherence, outpatient attendances, hospitalisations, cardiac rehabilitation)

#### Peer-reviewed studies

Thomas et al. examined the conditions accounting for attendances at a community-controlled Aboriginal Medical Service in Darwin [31]. HF was managed at 3.4% (CI 1.9-4.9%) of consultations, by an Aboriginal health worker (AHW) only (42.6%), an AHW together with a doctor (53.5%) or a doctor alone (3.9%). No non-Indigenous comparison group was examined in the study. However, the authors noted that in national data from the Australian Morbidity and Treatment Survey, HF had been managed at 1.9% of primary care (i.e., general practice) consultations. Another study from NSW, reported in a conference abstract, found that of 68 patients referred to an urban AMS specialist cardiology clinic, 3% had heart failure [30].

#### Other reports

Between 1999 and 2011, six government reports were published providing data on differences in hospital separation rates for HF between indigenous and non-Indigenous people in various Australians jurisdictions. These reports were based on data derived from the National Hospital Morbidity Database (NHMD), and were characterised by separation-based rather than person-based analyses and incomplete (and varying) coverage of all states and territories (due to poor quality of data in some jurisdictions).

A Commonwealth Department of Health and Aged Care report (1999) estimated a total of 970 Indigenous and 39,305 non-Indigenous public and private hospital separations in Australia with an ICD-9-CM principal diagnosis code for heart failure. Neither crude nor standardised separation rate ratios were provided [37].

The 2003 AIHW report *Heart failure…what of the future?* analysed NHMD data from South Australia and the Northern Territory only (the NT providing public hospital data only) for two time periods, 1995-96/1997-98 and 1998-99/2000-01. Although limited in geographic and age (45 years and over) coverage, a fall in HF hospitalisation rates was reported for male and female Indigenous as well as non-Indigenous populations. In the later triennium, age-standardised hospitalisation rates where heart failure or hypertensive heart disease was the principal diagnosis were 1555 per 100,000 for the male Indigenous population (compared with 743 for the male non-Indigenous population) and 1579 per 100,000 for the female Indigenous population (compared with 541 per 100,000 in the female non-Indigenous population). This translates into a standardised rate ratio of about 2 for men and 3 for women. As with mortality rates, hospitalisation rates for Indigenous males were higher in the 55–64 year age group than older age groups [34].

Australian hospital separations (excluding Tasmania, ACT and private hospitals in NT) from July 2004 to June 2006 were also reported by the AIHW, by Indigenous status, for the top 10 ambulatory care sensitive conditions [35]. For congestive heart failure, age standardised rates were 6.6 separations per 1000 Indigenous people (95% CI 6.3-6.9), compared with 1.9 per 1000 for ‘Other’ persons, reflecting a standardised separation ratio of 3.4. Analogous data were published for the period July 2006 to June 2008. Age standardised rates were 5.9 separations per 1000 Indigenous people (95% CI 5.6-6.1), compared with 2.0 per 1000 for ‘Other’ persons (Ratio 3) [36].

More recently, a Productivity Commission report provided multijurisdictional National Hospital Morbidity Data on 2008–2009 hospital separations for selected chronic conditions by Indigenous status [38]. Tasmania, ACT and private hospitals in the NT did not contribute data. For congestive heart failure, the rates of separations per 1000 people, directly standardised using the Australian 2001 standard population, were 6.1 and 2.0 for patients identified as Indigenous and non-Indigenous respectively.

Additionally, the AIHW published 2008–2009 public and private hospital separation rates for all states and territories combined, by Indigenous status, in which the principal diagnosis was a condition for which hospitalisations are considered potentially preventable, including congestive heart failure [39]. The crude rates, adjusted in the original report for Indigenous under-identification, were 2.8 per 1000 population for Indigenous persons and 2.1 per 1000 for non-Indigenous persons (Crude rate ratio = 1.33).

Despite differences in methodology with respect to age group inclusion, geographic coverage and diagnostic codes, these government reports show that Indigenous people in Australia have about 3 times higher hospitalisation rates than non-Indigenous people, when the different age distribution is taken into account. Average bed days per HF admission were lower in Indigenous compared with non-Indigenous patients [35,37].

The Bureau of Health Information in NSW has recently published two reports based on administrative data from that state [40,41]. The first examined patient admissions to public and private hospitals, and reported that 2% of the “potentially avoidable” admissions of patients with HF occurred among Indigenous people, the same as the population proportion of Indigenous people in NSW [40]. This study reported crude proportions only (and thus did not take into account age differences) and was not person-based, hence could not identify recurrent admissions of the same person. The second report linked hospitalisation and mortality data in a cohort of individuals aged >45 years with evidence from hospital records of pre-existing HF. Aboriginal people were over-represented among those who had more than one admission with HF during the year of follow-up: 3% were Aboriginal compared to 2% among those with one or no HF admissions [41]. While this study used person-based data, the proportion of the cohort identified as Aboriginal was not stated, and no adjustment was made for Indigenous under-identification. Thus, while the reports overall suggested by crude comparison that potentially avoidable admissions for HF occurred no more frequently among Indigenous compared with non-Indigenous people [40], recurrent admissions were more frequent in Indigenous people [41]. Additionally, the exclusion of people aged <45 years excludes a substantial proportion of Aboriginal people at risk for HF due to high rates of IHD and RHD at younger ages.

The only reports covering primary care were the *Bettering the Evaluation and Care of Health* (BEACH) surveys, conducted periodically by the AIHW Australian General Practice (GP) Statistics and Classification Unit. These provide data on GP encounters (~1,000 GPs nationwide participate annually, with data on 100 consecutive encounters collected from each). Data from the BEACH survey on Indigenous HF have been reported in two AIHW publications. For the BEACH survey years 2002–03 to 2006–07 inclusive, the reported crude proportion of encounters (number per 100) at which HF issues were managed by GPs was 1.0 (95% CI 0.6-1.3) for Indigenous patients vs. 0.7 (95% CI 0.7-0.8) for ‘Other’ patients, with an age-standardised rate ratio of 2.7 for Indigenous versus ‘Other’ patients [35]. For the overlapping period April 2004-March 2005 to April 2008-March 2009 inclusive, the reported crude proportion of encounters at which HF issues were managed by GPs was 0.9 (95% CI 0.6-1.2) for Indigenous patients vs. 0.7 (95% CI 0.7-0.7) for ‘Other’ patients, with an age-standardised rate ratio of 2.6 for Indigenous versus ‘Other’ patients [36].

### Health service delivery issues (including needs, access and barriers)

In a geo-mapping study of national HF services, Clark et al. reported lower service provision in rural and remote areas [32]. The spatial distribution of population, which included Indigenous status, was derived from Australian Bureau of Statistics Census data. HF prevalence was not measured directly but instead derived from European estimates, with Indigenous prevalence-weighting applied to communities, based on a combination of census data and AIHW-derived HF prevalence estimates.

Aspin and colleagues have recently reported findings from a qualitative study in which patients with chronic diseases (HF, diabetes and chronic obstructive pulmonary disease) were interviewed about the barriers and facilitators of access to health care and support [42]. Participants were recruited from Aboriginal Medical Services. Eleven of the 19 people interviewed had HF, with or without the other conditions. Those with HF were not distinguished from those with other chronic diseases, but issues were considered to be common across all the diseases. The culturally inappropriate services, racism, poor communication with health professionals and financial barriers were all impediments to service access, whereas support from family, the strength drawn from the Aboriginal community and regular access to good primary health care were regarded as assisting with participation in care.

### Costs related to HF diagnosis and care

No peer-reviewed studies were identified on the absolute or relative costs of HF management among Indigenous Australians. In 2011, the AIHW published expenditure estimates based on National Hospital Morbidity Data on potentially preventable hospital separations, by Indigenous status, for both public and private hospitals in all states and territories during the period 2008–09. Patients identified as Indigenous accounted for 3.9% of the expenditure for HF overall ($14.5 million from a total of $372.8 million for all patients). This corresponded with a calculated approximate expenditure on HF hospitalisation of $26.70 per Indigenous person, compared with $16.90 per non-Indigenous person (crude Indigenous to non-Indigenous expenditure ratio =1.58) [39]. In the analysis, a loading of 5% was added to the Indigenous patient costs to account for previously estimated excesses in comorbidity for similar Diagnosis Related Groups.

## Discussion

This comprehensive review of HF among Indigenous Australians reveals substantial current knowledge gaps that potentially hinder health service planning. Peer-reviewed journal papers as well as other publications sourced from administrative data were considered for inclusion, which was determined by relevance to a range of pre-defined sub-headings under the broad rubric of HF.

Priority was given to peer-reviewed articles. An exhaustive, multiple-database keyword search strategy was used for the journal literature, maximising the sensitivity of citation retrieval. However, most papers identified were either not principally concerned with HF and/or provided local data with questionable generalisability. Despite abundant published research on the likely major antecedents of HF in this population (coronary disease, hypertension and rheumatic heart disease), few articles included explicit mention of HF, an essential inclusion criterion.

The search of applicable Australian government websites as well as the Google interface identified a number of reports that provided data comparing Indigenous with non-Indigenous populations; these dominated the publications identified. Web searches of this type are inherently less systematic than those of journal databases, and some grey literature may have been overlooked. The validity of government reports is compromised by the quality of administrative data with respect to ascertainment of HF, the accuracy of which varies depending on the indicator being measured [43,44] and by shortcomings in the identification of Indigenous status. Additionally, these data capture only hospitalisations and deaths, not the less severe end of the illness spectrum [45,46]. Despite these limitations, the indicators show substantial disparities in the occurrence of HF between Indigenous and non-Indigenous populations.

The only population-based Australia-wide estimate of prevalence of HF among Indigenous Australians identified in our search was derived from the 2004–05 NATSIHS survey [33]. An age-standardised prevalence of HF 1.7 times higher among Indigenous than non-Indigenous Australians was reported, but the precision of estimates (judging by wide confidence intervals) was poor, particularly for Indigenous males. Although the ABS made substantial efforts to optimise Indigenous participation and the accuracy of self-report in the survey, the latter was compromised by the potential for differential ascertainment of HF from Indigenous and non-Indigenous subjects and the conflation of non-specific ‘oedema’ with HF. The recent cross-sectional population-based study in several Central Australian communities, incorporating comprehensive cardiovascular assessment [25], detected HF in 5.3% (95%CI 3.2-7.5%) of study participants, with only 35% of these having a pre-existing diagnosis of this condition. Although interpretability is limited by the absence of a non-Indigenous comparison group and by uncertainty regarding the representativeness of the participants, making it difficult to compare with whole-population data from the NATSIHS survey [33], there appears to be considerable under-diagnosis, at least in remote areas. The high proportion of newly detected cases in a young population (mean age 44 years [SD14]) raises the possibility of a ‘large, as yet unidentified, burden of HF in the [broader] Australian Aboriginal population’ [25].

The higher prevalence of HF in Indigenous people is also seen in clinical populations, with two retrospective cohort studies in which HF was a significantly more common current or previously documented co-morbidity in Indigenous compared to non-Indigenous patients hospitalised for myocardial infarction [23,24]. Death data in government reports are an important although problematic source of information about HF among both Aboriginal and non-Aboriginal people. The AIHW publications with multi-jurisdictional mortality data report death rates due to HF in the Indigenous population as double to treble those of non-Indigenous Australians, with the ratio higher for the middle aged. However, analysis of deaths attributed to HF is inherently problematic, particularly from administrative data. It has been argued that HF is a mode rather than a cause of death, that it is inconsistently recorded in death certification, and that the redistribution from HF codes to other causes in death coding is poorly standardised to the underlying aetiology [47]. Indeed, deaths reported as HF-related in administrative data have been considered as constituting ‘garbage’ codes that obscure the epidemiology of underlying causes of cardiovascular mortality [43]. It is also unknown to what extent comparative mortality rates have been miscalculated because of misclassification of Indigenous status, although linked data studies indicate that Indigenous all-cause mortality rates are underestimated [48].

In the literature reviewed, HF was frequently mentioned in passing as a subsidiary endpoint or as a covariate in analysis without further elaboration or interrogation. Not unexpectedly, HF was often a concomitant or complication of coronary disease [23,24,28], rheumatic heart disease [29], and, in one report, chronic airway disease (bronchiectasis) [26]. Finding that the proportion of Indigenous HF in which ejection fraction is maintained was at the upper limit of that expected from studies of other populations [27] is consistent with a high prevalence of coronary and hypertensive heart disease and ‘diabesity’ in this population [5]. However, no studies were identified that specifically provided data on the distribution of underlying causes of HF in the Indigenous population nationwide, although the most recent Australian and Indigenous Burden of Disease reports describe the use of (unpublished) estimates from hospital data to redistribute the burden of HF to underlying causes [45,49]. Heart failure in a Central Australian adult Aboriginal population sample was strongly associated with well-recognised risk factors (most especially coronary disease, diabetes mellitus, hypertension, obesity and rheumatic heart disease or history of rheumatic fever) [25].

Consistent with international evidence [50], HF is a predictor of increased morbidity and mortality following myocardial infarction in both Indigenous and non-Indigenous Australians [24]. Nonetheless, there are no longitudinal follow-up data quantifying or examining the determinants of morbidity, quality of life, survival or mortality among Indigenous Australians with established HF. Given the rather bleak natural history of HF regardless of cause [15] and the known efficacy of timely interventions [16,17], optimal tertiary prevention tailored to the needs of the Indigenous HF population is likely to be useful in improving Indigenous outcomes.

The three-fold excess frequency of HF-related hospitalisations reported by AIHW for Indigenous Australians parallels the estimated disparity in death rates. Administrative data on hospitalisation are widely used for information about chronic illnesses such as HF, but are influenced by factors such as access to and quality of ambulatory care as well as underlying disease prevalence and severity [51]. Further limitations include the inter-jurisdictional variation in quality of Indigenous identification [35,36], the shortcomings of HF ascertainment from disease codes [18,44], and their inability to capture information on disease frequency in the community. In metropolitan settings, a HF-related code in administrative data indicates a clinically verifiable diagnosis of HF with a positive predictive value of >90% [46]. However, it is unknown to what extent this validation is applicable to rural and remote settings where access to specialist expertise and diagnostic technology is more limited and the proportion of Indigenous patients is generally higher. The sensitivity of HF detection in administrative records can be very poor, even in urban teaching hospitals [18].

No report identified for this review combined both age-standardised analysis and investigation of Indigenous identification, both of which are critical for interpretability. One report comparing Indigenous and non-Indigenous hospitalisations for HF excluded persons <45 years, the age range in which disparities appear to be the greatest [34]. The markedly lower hospitalisation rate ratio in crude data (1.33) compared to about 3 in age-standardised comparisons [39] highlights the differing age distributions of the populations and the early age of HF onset in the Aboriginal population. Meaningful comparison of Indigenous and non-Indigenous HF-related hospitalisations requires inclusion of adults across a wider age range. There is an interaction of Aboriginality with age, therefore a single age-standardised rate over all adult ages will obscure variation in disparities across the lifespan. This can be addressed by comparing age-standardised rates separately for younger and older age groups as has been done in a study of Indigenous incidence rates for myocardial infarction [52].

The listing by AIHW of HF as among ‘preventable’ causes of hospitalisation highlights the role of primary care in preventing hospital admissions [51]. Only one peer-reviewed article reported general practice attendances by Indigenous Australians for HF management, based on attendances at a Darwin AMS [31], and one conference abstract reported attendances at a cardiology clinic within an AMS in Sydney [30]. Neither study included local non-Indigenous comparison data. Their generalisability to Indigenous populations elsewhere is also uncertain. The BEACH survey showed an age-standardised proportion of GP visits for HF as 2.6-fold higher among Indigenous than ‘Other’ attendees. However, a sub-study in which Indigenous identification was specifically explored suggested considerable Indigenous under-identification with 2.2% of patients in the sub-study identified as Indigenous, compared with 1.4% in the main survey [36]. Furthermore, it is difficult to interpret data on condition-specific proportions of health encounters such as primary care attendances, as they are not individual patient-based and depend on both the frequency of encounters for other conditions and variation in the condition-specific expertise offered by different providers. Between-population comparisons of such proportions are thus confounded by other morbidities, and cannot be taken at face value as indicators of disease frequency or disease-specific health behaviour.

As cardiovascular disease is largely preventable and linked to social determinants of health, these should be an important focus for health care interventions. Access to health care is an important moderator for health outcomes. Accordingly, in addition to epidemiological indicators, affordability, cultural appropriateness and geographical access to health services need to be investigated. Although the complex needs and many barriers that Indigenous people experience in accessing health services are well documented [28,53,54], only one publication was identified that had a strong focus on HF in this regard [42]. Late presentation is cited as a contributor to poor health outcomes for Indigenous Australians, such as in the setting of acute coronary syndrome [55], but there are no published data for this population on delayed in presentation in HF specifically. The work of Aspin and colleagues [42] accords with the barriers and inequities already described for ACS, cancer, renal and mental health. Common themes include negative associations with hospitals as a result of death of relatives, racism and cultural alienation; social, economic and cultural barriers; and competing priorities [56,57]. However, given the complexity of HF, further research could usefully identify specific HF-related issues in order to optimise its culturally appropriate management, including palliative care, in this population.

The overall lack of data on HF therapeutics in the Indigenous population is also noteworthy. Management interventions for HF often entail complexity that is particularly challenging for individuals in disadvantaged and remote communities. For example, chronic anticoagulation is problematic, given the requirement for rigorous monitoring to avoid potentially life-threatening adverse effects [58]. Disease management is impacted by the disproportionate burden of co-morbidities that accompanies HF in this population, with co-existing conditions being important determinants of quality of life, the complexity of medical interventions, and survival. Furthermore, biological differences between population groups in response to HF therapies have been noted in other settings [59]. This issue remains unexplored for the Australian Indigenous population.

## Conclusions

The poor health outcomes experienced by Indigenous Australians can be attributed to socio-economic disadvantage and marginalisation and are manifest in a range of chronic conditions that include cardiovascular diseases [60]. Heart failure, a disabling and survival-limiting ‘downstream’ complication of these conditions, presents substantial challenges in its own right. Although this systematic review demonstrates that high-quality data are limited, better information is beginning to emerge, bolstering the evidence that Indigenous Australians have an excess burden of heart failure in comparison with their non-Indigenous counterparts.

Clearly, optimising the delivery of preventive and therapeutic interventions for HF in Indigenous Australians is predicated on a sound knowledge of the underlying causes and comorbidity burden. The likely antecedents and concomitants of HF in this population are well described: all elements of the cluster of hypertension, coronary heart disease, chronic kidney disease, metabolic syndrome and diabetes are known to be prevalent in substantial excess [6,7,10]. The substantial proportion of HF cases with preserved ejection fraction documented in North Queensland [27] and Central Australia [25] is consistent with this aetiological spectrum. Additionally, the prevalence of rheumatic valvular disease is extremely high among certain Indigenous populations, namely those in Northern Australia [61]. However, the role of these conditions in the pathogenesis of HF in Indigenous Australians has only been specifically examined in a single Central Australian study and its generalisability to the broader Australian context is uncertain [25]. The aetiological contribution of these risk factors to the burden of HF in the greater Australian Aboriginal population has not been formally quantified and constitutes an important gap in current knowledge.

Accurate data on indicators of HF among Indigenous Australians, as well as information on their access to and utilisation of health services for this problem, could inform better care and policy development but are limited by the scarcity of quality information identified in this review. Considering the constraints on administrative data, high-quality research is required to confirm epidemiological indicators of HF and to monitor trends adequately. Such data are now beginning to emerge [25] and reflect growing recognition of the huge health disparities in Indigenous Australians and commitment to Closing the Gap. The Australian Bureau of Statistics has publicised that the next NATSIHS will be the largest Aboriginal and Torres Strait Islander health survey to date and ‘will expand on the 2004–05 survey by increasing the number of participants by 30%, collecting new information on exercise, diet (including bush foods) and measures of cholesterol, blood glucose and iron. For the first time, the ABS will directly measure obesity and blood pressure levels, as well as nutritional status and chronic disease’ [62]. In view of the diversity of Indigenous populations and known geographical inequities in health service provision, local as well as whole or multi-jurisdictional epidemiological studies are desirable. In addition, qualitative investigations of the impact of HF and effective, culturally suitable approaches to HF management in Indigenous Australians are needed.

## Abbreviations

ABS: Australian Bureau of Statistics; ACS: Acute coronary syndrome; AIHW: Australian Institute of Health and Welfare; AMS: Aboriginal Medical Service; ARF: Acute rheumatic fever; BEACH: Bettering the Evaluation and Care of Health Survey; HF: Heart failure; NATSIHS: National Aboriginal and Torres Strait Islander Health Survey; NHMD: National Hospital Morbidity Database; NMD: National Mortality Database; RHD: Rheumatic heart disease.

## Competing interests

The authors declare that they have no competing interests.

## Authors’ contributions

All authors contributed to the conception and design of the study, participated in writing the manuscript, and approved the final draft. JW undertook the literature search and retrieval of publications. JW and JK reviewed the retrieved publications for inclusion in the study.

## Pre-publication history

The pre-publication history for this paper can be accessed here:

http://www.biomedcentral.com/1471-2261/12/99/prepub

## Supplementary Material

Addtional file 1 **Table S1.** Search Terms. Click here for file
