# Supplementary material for: Heart failure among Indigenous Australians: a systematic review
Source: BMC Cardiovasc Disord. 2012 Nov 1;12:99. doi: 10.1186/1471-2261-12-99 (PMC3521206; doi:10.1186/1471-2261-12-99)
Supplement: Addtional file 1 — Table S1. Search Terms. [file 1471-2261-12-99-S1.doc]

Supplementary Table: Search Terms

| (Health services, indigenous [mesh] OR oceanic Ancestry Group [mesh] OR aborigin* OR indigeno* OR indigene*)  AND  (cardiac output, low [mesh] OR cardiomegaly [mesh] OR cardiomyopathies [mesh] OR heart failure [mesh] OR ventricular dysfunction [mesh] OR “heart failure” OR “cardiac failure” OR “ventricular dysfunction” OR “systolic function” OR “diastolic function” OR “systolic dysfunction” OR “diastolic dysfunction” OR “systolic impairment” OR “diastolic impairment” OR cardiomegaly OR cardiomyopath* OR “backward failure” OR “cardiac decompensation” OR “cardiac incompetence” OR “cardiac insufficiency” OR “cardial decompensation” OR “cardial insufficiency” OR “heart insufficiency” OR “decompensatio cordis” OR “heart decompensation” OR “heart incompetence” OR “heart insufficiency” OR “insufficientia cardis” OR “myocardial failure” OR “myocardial insufficiency” OR “cardiopulmonary insufficiency” OR “forward failure” OR “ventricle failure” OR “ventricular failure” OR “ventricle overload” OR “ventricular overload” OR “heart myopathy” OR myocardiopath* OR “primary myocardial disease”)  The truncation symbol (* in PubMed) was modified according the requirements of each electronic database |
| --- |
